# Successful determination of nilotinib dosage by therapeutic drug monitoring in a patient with chronic myeloid leukemia developing hepatic dysfunction: A case report

**DOI:** 10.1002/ccr3.2191

**Published:** 2019-06-14

**Authors:** Ryosuke Nakahara, Takahiro Sumimoto, Masao Ogata, Yuhki Sato, Hiroki Itoh

**Affiliations:** ^1^ Department of Clinical Pharmacy Oita University Hospital Yufu‐shi Japan; ^2^ Department of Hematology Oita University Hospital Yufu‐shi Japan

**Keywords:** chronic myeloid leukemia, hepatic dysfunction, nilotinib, therapeutic drug monitoring

## Abstract

Fixed dosage regimen is currently the standard therapy with tyrosine kinase inhibitors (TKI). This case report demonstrates successful determination of nilotinib dosage by therapeutic drug monitoring (TDM) in a patient with chronic myeloid leukemia (CML). TDM may provide useful marker for individualized dosing of TKI for the treatment of CML.

## 1 INTRODUCTION

Nilotinib is a second‐generation BCR‐ABL tyrosine kinase inhibitor (TKI) and has antileukemic activity against chronic myeloid leukemia (CML). Randomized phase 3 trials have revealed that nilotinib and dasatinib, second‐generation tyrosine kinase inhibitors (2nd TKIs) of BCR‐ABL, have superior efficacy compared with imatinib for the first‐line treatment of chronic myelogenous leukemia in the chronic phase (CML‐CP).^1^, ^2^ Currently, both these 2nd TKIs are available as the first‐line treatment in newly diagnosed CML‐CP. Their profiles of adverse events are characteristic. Typical adverse events of nilotinib include hepatic dysfunction, elevated bilirubin, prolongation of QTc interval, hyperlipidemia, and hyperglycemia. Hepatotoxicity caused by other tyrosine kinase inhibitors is known, but it is reported that the frequency of nilotinib hepatotoxicity is rare.^3^ When hepatic dysfunction occurs, drug withdrawal or dose reduction is required, but there are currently no indicators for dose adjustment. In this case report, we describe a case of successful determination of nilotinib dosage by therapeutic drug monitoring (TDM) in a CML patient who developed hepatic dysfunction during nilotinib therapy.

## 2 CASE PRESENTATION

**Figure 1:**
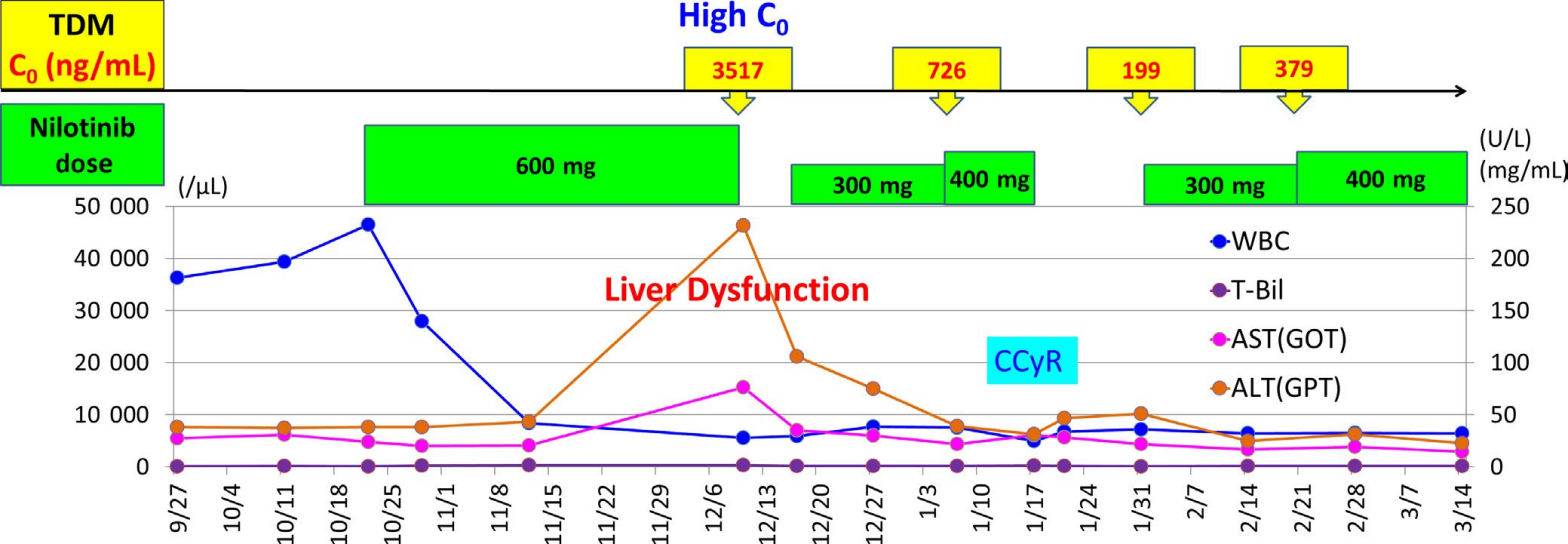
Clinical course. Discontinuation of nilotinib and restarting with a lower dose succeeded to adjust plasma concentration of nilotinib to optimal level and normalize hepatic function. White blood cell count decreased to baseline level (3300‐8600/μL), then reached CCyR. ALT, alanine transaminase; AST, aspartate transaminase; C_0_, trough concentration; CCyR, complete cytogenetic response; GOT, glutamic oxaloacetic transaminase; GPT, glutamic pyruvic transaminase; T‐Bil, total bilirubin; TDM, therapeutic drug monitoring; WBC, white blood cell count. *X*‐axis on the left represents WBC count (/μL). *Y*‐axis on the right represents AST (U/L), ALT (U/L) and T‐Bil levels (mg/dL)

A 76‐year‐old man presented at our hospital with an abnormal increase in white blood cell count (WBC) during regular follow‐up after prior renal cell carcinoma surgery. His history was only mild hypertension, and there was no hepatic disease like chronic hepatitis such as hepatitis B or C. In addition, there was almost no drinking history with one beer of 350 mL a week. On September 27, 2012, his white blood cell count (WBC) was elevated to 36,200/μL, and he was clinically diagnosed with chronic phase Philadelphia‐positive CML. On October 10, 2012, blood tests, bone marrow examination, and imaging findings confirmed CML. Initially, nilotinib was administered to the patient at a dose of 600 mg twice a day (BID). Two months after nilotinib administration, hepatic dysfunction (grade 3) was observed. At that time, plasma concentration of nilotinib was determined by a high‐performance liquid chromatographic method as described previously.^4^ Using this method, the trough plasma concentration of nilotinib was 3517 ng/mL (Figure 1). This value was markedly higher than the mean trough concentration (615 ng/mL) reported in a phase I/II trial.^5^ Due to hepatic dysfunction and elevated plasma nilotinib concentration, nilotinib was discontinued on December 10. On December 17, hepatic function was improved and nilotinib was restarted at a lower dose of 300 mg BID. Twenty days later, plasma nilotinib concentration was 726 ng/mL, which was significantly lower than the previous level and close to the reported mean trough concentration (615 ng/mL).^5^ On January 17, 2013, the proportion of Philadelphia chromosome‐positive cells in bone marrow was 0%, and complete cytogenetic response was achieved. Thereafter, even when nilotinib was suspended due to influenza infection, TDM was utilized at the time of drug resumption and dosage adjustment. On March 14, major molecular response (BCR‐ABL^IS^: international scale ≤0.1%) was achieved without any adverse event. Eleven months after the start of nilotinib therapy, complete molecular response (BCR‐ABL^IS^≤0.0032%) was achieved. The treatment was continued until January 2018. Since then, the attending physician decided to stop nilotinib and followed up, because the patient was remained in remission for more than 4 years after achieving complete molecular response. There has been no disease progression, and his condition is stable.

## 3 DISCUSSION

Nilotinib is a second‐generation BCR‐ABL TKI and has antileukemic activity against CML. Treatment of CML has improved dramatically with the development of TKIs. However, the inter‐individual variability in adverse events and clinical efficacy as well as high drug cost remain major issues and present a major obstacle to treatment. Therefore, TDM of TKIs is an important tool for CML treatment. The safety and efficacy of nilotinib have been reported in previous clinical trials.^6^, ^7^, ^8^, ^9^ To ensure an optimal trough plasma concentration of nilotinib is important for ensuring maximum efficacy in patients with imatinib‐resistant or imatinib‐intolerant CML.^5^ However, in nilotinib therapy, there is no case report of effective dose adjustment using TDM at the onset of adverse events. In the present report, when hepatic dysfunction occurred after initiation of nilotinib therapy, TDM revealed markedly elevated plasma concentration of nilotinib (3517 ng/mL). Accordingly, the dosage of nilotinib was reduced. As a result, plasma concentration of nilotinib was reduced to 726 ng/mL which approached the reported mean trough level, and WBC count decreased with achievement of major molecular response. This result also supports previous report that the target trough concentration of nilotinib is 800 ng/mL.^9^ Subsequently, TDM was used to guide dose adjustment, resulting in stabilized liver function and eventually complete cytogenetic and molecular responses. Thus, TDM allowed maintenance of optimal plasma nilotinib concentrations, which not only prevented the occurrence of adverse events, but also maintained clinical efficacy.

Recent study has reported that hepatic dysfunction tends to occur if the plasma trough concentration of nilotinib is high.^10^ Despite the fact that pharmacokinetic exposure of TKIs is highly variable and a clear relationship exists between exposure and treatment outcomes, fixed dosing is still standard practice. This case report demonstrates successful determination of nilotinib dosage by TDM in a CML patient. TDM may provide useful marker for individualized dosing of BCR‐ABL TKIs for treating CML.

## CONFLICT OF INTEREST

There are no conflicts of interest to report.

Patient consent: Written informed consent was obtained from the patient for publication of this case report and accompanying clinical data.

## AUTHOR CONTRIBUTION

RN: analyzed data and wrote the manuscript. TS: analyzed data. MO: managed the patient. YS: wrote the manuscript and reviewed the manuscript. HI: reviewed the manuscript. All authors read and approved the manuscript to be submitted.
